# Illness representations, psychological distress and non-cardiac chest pain in patients attending an emergency department

**DOI:** 10.1080/08870446.2014.923885

**Published:** 2014-08-07

**Authors:** R. Webster, P. Norman, S. Goodacre, A.R. Thompson, R.R.C. McEachan

**Affiliations:** ^a^ Department of Psychology, University of Sheffield, Sheffield, UK; ^b^ School of Health and Related Research, University of Sheffield, Sheffield, UK; ^c^ Clinical Psychology Unit, Department of Psychology, University of Sheffield, Sheffield, UK; ^d^ e-Health Unit, Research Department of Primary Care and Population Health, University College London, London, UK; ^e^ Bradford Institute for Health Research, Bradford Teaching Hopsitals NHS Foundation Trust, Bradford Royal Infirmary, Bradford, UK

**Keywords:** non-cardiac chest pain, anxiety, depression, quality of life, illness representation, psychosomatic

## Abstract

***Objective:*** Many patients who attend an emergency department (ED) with chest pain receive a diagnosis of non-cardiac chest pain (NCCP), and often suffer poor psychological outcomes and continued pain. This study assessed the role of illness representations in explaining psychological distress and continued chest pain in patients attending an ED.

***Methods:*** ED NCCP patients (*N* = 138) completed measures assessing illness representations, anxiety, depression and quality of life (QoL) at baseline, and chest pain at one month.

***Results:*** Illness representations explained significant amounts of the variance in anxiety (Adj. *R*² = .38), depression (Adj. *R*² = .18) and mental QoL (Adj. *R*² = .36). A belief in psychological causes had the strongest associations with outcomes. At one month, 28.7% of participants reported experiencing frequent pain, 13.2% infrequent pain and 58.1% no pain. Anxiety, depression and poor QoL, but not illness representations, were associated with continued chest pain.

***Conclusions:*** The findings suggest that (i) continued chest pain is related to psychological distress and poor QoL, (ii) interventions should be aimed at reducing psychological distress and improving QoL and (iii) given the associations between perceived psychological causes and psychological distress/QoL, NCCP patients in the ED might benefit from psychological therapies to manage their chest pain.

## Introduction

Acute chest pain accounts for approximately 700,000 emergency department (ED) attendances each year in England and Wales (Goodacre et al., 2005), but between 30 and 60% of these patients do not receive a cardiac diagnosis for their pain (Eken et al., 2010; Mayou & Thompson, 2002). Current guidelines recommend that staff merely explain the non-cardiac nature of the pain to these patients (National Institute for Health and Clinical Excellence, 2010), based on the assumption that the rapid diagnosis of non-cardiac chest pain (NCCP) will alleviate any distress. However, cardiac testing itself may lead some patients to believe they are suffering from a cardiac problem, and so the unclear diagnosis, along with a lack of follow-up, may cause psychological distress (Nijher, Weinman, Bass, & Chambers, 2001). Furthermore, reassurance that test results are negative is often not sufficient to calm patients’ concerns (McDonald, Daly, Jelinek, Panetta, & Gutman, 1996). Despite having excellent long-term cardiac survival (Papanicolaou et al., 1986), patients with NCCP have been found to experience elevated levels of anxiety, reduced quality of life (QoL), further episodes of chest pain, and high use of health care services (e.g. Eslick & Talley, 2008; Goodacre, Mason, Arnold, & Angelini, 2001; Hadlandsmyth, Rosenbaum, Craft, Gervino, & White, 2013; Smeijers et al., 2013; Webster, Norman, Goodacre, & Thompson, 2012), thus placing a burden on health care costs and resources (Eslick, Coulshed, & Talley, 2002). Furthermore, indirect economical costs may also result from chronic chest pain through lost work days (Eslick et al., 2002). Psychological morbidity associated with NCCP may initiate, maintain or worsen chest pain (Bass & Mayou, 2002; Potts & Bass, 1995). In particular, anxiety-related disorders are often proposed as a cause for NCCP (Beitman et al., 1987; Jonsbu et al., 2009), and higher levels of anxiety have been related to increased health care use in NCCP patients (Hadlandsmyth, Rosenbaum, Craft, Gervino, & White, 2013). These findings suggest that it may be important to address not only the NCCP, but also the psychological distress associated with it.

In order to develop effective interventions for this population, it is important to identify factors that are associated with both psychological distress and continued chest pain. However, research to date has focused on a limited range of mainly demographic variables and has lacked a strong theoretical focus (Webster et al., 2012). The common sense model of illness representations (CSM, Leventhal, Meyer, & Nerenz, 1980) provides an appropriate theoretical framework for examining the correlates of poor psychological outcomes in patients with NCCP (Webster et al., 2012). The CSM proposes that when faced with a health threat or illness, such as the experience of chest pain, people form a representation of the health threat based around the perceived causes of the illness, consequences of the illness, identity (i.e. the label given to the illness and the symptoms associated with it), expected timeline of the illness, cure/controllability of the illness (personal and treatment), one’s emotional response to the illness and one’s perceived understanding of the illness (coherence). Illness representations have been found to explain both psychological and physical outcomes for a range of conditions (e.g. Hagger & Orbell, 2003), including medically unexplained symptoms (MUS) (Frostholm et al., 2007) and cardiac chest pain (Aalto et al., 2006).

To date, four studies have applied the CSM to patients with NCCP. Robertson, Javed, Samani, and Khunti (2008) found that those with NCCP had more negative illness representations than those with cardiac chest pain. Donkin et al. (2006) found that illness representations (i.e. stronger illness identity, the perception of more severe consequences and a longer timeline, increased illness concern and stronger emotional representations) assessed at baseline were related to lower levels of reassurance following a negative exercise stress test. Jonsbu, Martinsen, Morken, Moum, and Dammen (2012) found that more negative illness representations assessed prior to cardiac stress testing were related to increased depression, worse QoL and chest pain, both prior to testing and six months later. Schroeder et al. (2012) found that more negative consequences, more chronic timeline, stronger illness identity, and greater concern and emotional representations were related to more severe chest pain prior to diagnostic testing. However, these studies suffer from a number of important limitations. First, the studies used the brief Illness Perceptions Questionnaire (IPQ, Broadbent, Petrie, Main, & Weinman, 2006), which only uses single items to measure each dimension. Second, none of these studies assessed perception of possible causes of NCCP. Given that NCCP patients do not receive a diagnosis, this dimension may be very important in understanding NCCP patients’ psychological reactions. Third, Jonsbu et al. did not assess anxiety. Given that anxiety has previously been shown to be strongly associated with NCCP (Smeijers et al., 2013; White, Craft, & Gervino, 2010), this may be an important factor to assess. Fourth, previous studies have employed cross-sectional designs (Schroeder et al., 2012) or have assessed patients pre-diagnosis (before they were aware that their pain was non-cardiac), which may have impacted on their responses (Jonsbu et al., 2012; Robertson et al., 2008; Schroeder et al., 2012). Fifth, previous studies have been conducted in outpatient departments (Donkin et al., 2006; Jonsbu et al., 2012; Robertson et al., 2008; Schroeder et al., 2012), and therefore the patients may differ from those who are examined in an ED.

The present study therefore sought to address the above limitations by using a full version of the IPQ to assess illness representations, including perceived causes, in patients recruited in an acute setting (ED) who have been informed of their NCCP diagnosis, and then relate these perceptions to a range of measures of psychological distress and continued chest pain at one-month follow-up. In line with the CSM (Leventhal et al., 1980) and previous research on NCCP (e.g. Jonsbu et al., 2012; Schroeder et al., 2012; Webster et al., 2012), it was hypothesised that (i) illness representations would be associated with levels of psychological distress and QoL at baseline (i.e. after being informed of a NCCP diagnosis in an ED) and that (ii) baseline illness representations, psychological distress and QoL would be related to chest pain at one month.

## Method

### Participants and procedure

Participants were recruited from an ED in a large teaching hospital in a city in the north of England, UK. According to departmental protocol (see Goodacre et al., 2001), patients admitted with chest pain underwent a period of 4–6 hours observation by doctors and specialist chest pain nurses, which include serial electrocardiogram testing, cardiac enzyme tests and an exercise tolerance test to rule out Myocardial Infarction and angina. Patients found to have cardiac pain were admitted or referred for further investigation. Patients for whom no cardiac cause was identified were informed that their chest pain was not caused by a cardiac problem. No other firm diagnosis was given to patients; however, patients were routinely given a brief leaflet by the chest pain nurses, which provided information on NCCP, including possible causes, and recommended lifestyle behaviours (e.g. exercise). NCCP patients were discharged directly from the ED.

The recruitment period ran from September 2010 to July 2011, during the hours of 9 am until 7 pm, 7 days a week. Potential participants were first approached by a member of the clinical staff before being introduced to the researcher. After obtaining informed consent, participants were given a baseline (post-diagnosis) questionnaire that was either completed in the clinic or returned in a freepost envelope. Participants were sent a follow-up questionnaire by post one month later. Participants who failed to return their questionnaires were contacted by phone after one week.

The inclusion criteria for this study were based on a previous study in the same ED (Arnold, Goodacre, Bath, & Price, 2009). Patients were considered eligible for the study if they were admitted to the ED with acute chest pain of suspected cardiac origin. They were excluded if they received a cardiac diagnosis, had known Coronary Heart Disease, were unable to comprehend written English or had other life threatening non-cardiac pathology. Participants under the age of 25 were also excluded, as these patients generally do not undergo cardiac investigations for their pain due to the very low risk of cardiac problems.

The study received full ethical approval from NHS South Yorkshire Research Ethics Committee.

### Measures

In addition to demographic information (see Table 1), participants completed a number of established measures:

**Table 1.** Participant details at baseline and one-month follow-up.

Variable		Baseline (*N* = 200)	One-month (*N* = 138)
		Mean (SD)	Mean (SD)
Age		54.74 (11.81)	56.58 (11.46)**
Age at leaving education		17.35 (4.70)	17.29 (3.46)
Hours worked/week		36.65 (13.94)	36.10 (11.30)
		*n* (%)	*n* (%)
Gender (male)		113 (56.5)	71 (51.4)**
Marital status	Married	130 (67.7)	90 (65.2)
	Single	27 (14.1)	17 (12.3)
	Widowed	11 (5.7)	12 (6.2)
	Cohabiting	6 (3.1)	5 (3.6)
	Separated	5 (2.6)	2 (1.4)
	Engaged	1 (0.5)	1 (0.7)
Employment status	Employed	119 (62.0)	79 (57.2)
	Retired	55 (28.6)	44 (31.9)
	Unemployed	12 (6.2)	6 (4.3)
	Homemaker	4 (2.1)	2 (1.4)
	Student	2 (0.9)	2 (1.4)

** *p *< .01.

#### Illness representations

Illness representations were assessed using the Illness Perceptions Questionnaire-Revised (IPQ-R, Moss-Morris et al., 2002). For the purposes of this study, the term ‘my illness’ was substituted for the term ‘my chest pain’, and cardiac and gastrointestinal causes were added to the list of potential causes. This is in line with recommendations that the measure may be adapted for specific conditions where appropriate (Moss-Morris et al., 2002). Dimensions assessed were identity, chronic timeline (6 items, *α* = .82), cyclical timeline (4 items, *α* = .78), consequences (6 items, *α* = .84), treatment control (5 items, *α* = .84), personal control (6 items, α = .84), emotional representations (6 items, *α* = .87), coherence (5 items, *α* = .96), risk causes (6 items, *α* = .74), psychological causes (6 items, *α* = .86), immunity causes (3 items, *α* = .62), chance causes (2 items, *α* = .24), cardiac causes (1 item) and gastrointestinal causes (1 item). For the identity dimension, the number of symptoms (e.g. breathlessness and dizziness) that participants attribute to their chest pain is summed (min. 0 to max. 10). All other items are scored on five-point response scales and averaged to provide a score for each dimension. Higher scores indicate belief in a stronger identity, a longer timeline, worse consequences, more control, more coherence, more negative emotions and a stronger belief in the individual causes. Internal reliabilities for the subscales were all acceptable to high (Cronbach’s *α*’s > .70), except for the immunity causes (*α* = .62) and chance causes subscales (*α* = .24). As the Cronbach’s *α* for the chance subscale was particularly low, only the first item from this two-item subscale was used to assess chance causes (i.e. ‘Chance or bad luck’).

#### Anxiety and depression

Anxiety and depression were assessed using the Hospital Anxiety and Depression Scale (HADS, Zigmond & Snaith, 1983). Scores of 7 or less on each subscale indicate normal levels of anxiety or depressive symptoms, scores of 8–10 indicate mild symptoms and scores of 11 or more indicate moderate symptoms (Zigmond & Snaith, 1983). Internal reliabilities for the anxiety (*α* = .89) and depression (*α* = .86) subscales were good.

#### Quality of life

QoL was assessed using the SF-12 (Ware, Kosinski, & Keller, 1996), a shortened version of the SF-36 (Ware, Snow, Kosinski, & Gandek, 1993) which is a well-established measure of health status. The measure consists of two subscales: Physical Component Summary (PCS) and the Mental Component Summary (MCS). Higher scores indicate better QoL.

#### Chest pain

At one-month follow-up, participants were asked to report whether they were still experiencing chest pain, and if so, the frequency of their pain. Participants were classified as experiencing no pain, infrequent pain (<weekly) or frequent pain (≥weekly) at follow-up.

## Results

### Participants

During the study period, 4514 patients attended the ED with chest pain and were referred for cardiac investigation. Of these patients, 3797 met at least one of the exclusion criteria, including cardiac chest pain (*n *= 1068), known CHD (*n *= 827), other life-threatening non-cardiac pathology (*n *= 135), unable to speak/read English (*n *= 51), and aged under 25 (*n *= 27), as well as unrecorded (*n *= 1689) reasons. A further 228 patients were not invited due to administrative reasons (e.g. staff were too busy). Overall, 489 patients were invited to participate in the study; 83 (17%) declined participation. Of the 406 patients who agreed to participate in the study, 392 opted to return the questionnaire via freepost envelope. Over the course of the study eight participants withdrew, one died and seven could not be contacted as they no longer lived at their contact address. Of the remaining 390 participants, 200 (51%) returned the initial baseline questionnaire, of whom 138 (69%) completed the second questionnaire. Details regarding the demographic profile of participants at time 1 and 2 can be found in Table 1.

Participants who responded to the one-month follow-up questionnaire were significantly older, *t*(197) = 3.46, *p *= .001, more likely to be female, *x²*(1, *N *= 199) = 7.42, *p *=* *.006, less anxious, *t*(195) = 2.47, *p *= .014, had lower perceptions of risk, *t*(182) = 3.23, *p *= .001 and psychological causes, *t*(182) = 2.36, *p *= .020, than non-responders.

### Descriptive statistics

Mean scores for all psychological and illness representation variables are reported in Table 2. Participants scored near or below the mid-point on all IPQ-R measures. More detailed analysis of the perceived causes revealed that gastrointestinal causes received the highest mean rating and were the most strongly endorsed (i.e. a score of ≥4.00), by 31.2% of the sample. All other causes, with the exception of chance causes (19.7%), were strongly endorsed by less than 10% of the sample. Considering levels of psychological distress, 36.8% of participants scored above the cut-off of 8 for mild anxiety symptoms, 22.8% for depressive symptoms; and 17.6% of participants scored above the cut-off of 11 for moderate anxiety symptoms, 9.6% for depressive symptoms. Levels of anxiety (see Table 2) were significantly higher than population norms (Hinz & Brähler, 2011) (*M *=* *4.7), *t*(135) = 5.64, *p *< .001; however, levels of depression were not, (*M *=* *4.7), *t*(135) = 0.65, *p *= .52. Mental QoL was significantly poorer than in those with minor medical complaints (Ware et al., 1996), (*M *=* *53.82), *t*(135) = 8.84, *p *< .001; however, physical QoL did not differ, (*M *=* *47.42), *t*(135) = 0.28, *p *= .78. At one-month follow-up, 39 (28.3%) participants reported experiencing further frequent chest pain, 18 (13.0%) reported infrequent chest pain and 81 (58.7%) reported no further chest pain.

**Table 2.** Means, standard deviations and correlations between illness representations and outcome variables at baseline (*N *= 138).

Variable	Mean (SD)	Anxiety	Depression	Mental QoL	Physical QoL
Identity	1.54 (2.01)	.16*	.21**	−.13	−.24**
Timeline – chronic	2.21 (0.76)	.37***	.33**	−.31***	−.21**
Timeline – cyclical	3.02 (0.94)	.21**	.14	−.11	−.14
Consequences	2.31 (0.87)	.37***	.35***	−.37***	−.17*
Personal control	3.27 (0.91)	−.09	−.13	.09	.07
Treatment control	3.50 (0.77)	−.13	−.16*	.12	.01
Coherence	2.67 (1.15)	−.12	−.16*	.14	.08
Emotional representations	2.96 (0.93)	.49***	.39***	−.50***	−.12
Risk causes	2.42 (0.69)	.36***	.27***	−.31***	−.06
Psychological causes	2.60 (0.92)	.61***	.45***	−.61***	.01
Immunity causes	2.23 (0.79)	.16*	.15*	−.19*	−.21**
Chance causes	2.47 (1.15)	.05	.02	−.08	−.02
Cardiac causes	2.10 (1.03)	.24**	.15*	−.17*	−.05
Gastrointestinal causes	2.73 (1.27)	−.03	.02	.09	−.13
*M* (SD)		6.72 (4.19)	4.48 (4.03)	44.94 (11.71)	47.65 (9.54)

* *p *< .05

** *p *< .01

*** *p *< .001.

**Table 3.** Correlations between illness representation variables (*N *= 138).

Variable	Gastrointestinal causes	Cardiac causes	Chance causes	Immunity causes	Psychological causes	Risk causes	Emotional representations	Coherence	Treatment control	Personal control	Consequences	Timeline - cyclical	Timeline - chronic
Identity	−.21*	−.01	.03	−.00	.04	−.02	.09	−.09	.01	−.12	.24**	.01	.19*
Timeline – chronic	.08	.50***	.32***	.25**	.36***	.35***	.52***	−.31***	−.55***	−.44***	.63***	.30***	
Timeline – cyclical	.35**	.32***	.14	.28**	.22*	.35***	−.28**	−.28**	−.19*	−.16	.22*		
Consequences	.04	.43***	.31***	.27**	.46***	.40***	.50***	−.28**	−.40***	−.27**			
Personal control	−.09	−.36***	−.20*	−.16	−.04	−.11	−.20*	.55***	.74***				
Treatment control	.01	−.44***	−.23*	−.28**	−.11	−.22*	−.30**	.62***					
Coherence	−.07	−.44***	−.13	−.32***	−.12	−.27**	−.33***						
Emotional representations	.03	.37***	.16	.26**	.50***	.40***							
Risk causes	.29**	.49***	.25**	.51***	.54***								
Psychological causes	−.02	.31***	.10	.24**									
Immunity causes	.15	.30**	.30**										
Chance causes	.19*	.23*											
Cardiac causes	.19*												

* *p *< .05

** *p *< .01

*** *p *< .001.

### Associations between illness representations, psychological distress and QoL after diagnosis

Considering the IPQ-R dimensions, chronic timeline, consequences, emotional representations and perceived causes (risk, psychological, immunity, cardiac) were significantly correlated with anxiety, depression and mental QoL (see Table 2). In addition, identity was correlated with anxiety and depression, cyclical timeline with anxiety and treatment control with depression. Identity, chronic timeline, consequences and immunity causes were also correlated with physical QoL. Only personal control, chance causes and gastrointestinal causes were not correlated with any of the outcome variables. Younger age was associated with higher levels of anxiety, *r*(135) = −.23, *p *=* *.01, depression, *r*(135*)* = −.18, *p *=* *.04 and worse mental QoL, *r*(135) = −.20, *p *=* *.02; gender was not related to any of the psychological outcomes.

A series of regression analyses were performed to examine the extent to which illness representations explained variance in baseline psychological distress and QoL. Separate analyses were conducted for each of the psychological distress and QoL measures as dependent variables (i.e. anxiety, depression, mental QoL and physical QoL). As age correlated with most of the outcome variables, it was included as an independent variable, along with all illness representation variables. Beta values for each analysis are reported in Table 4. For each analysis, plots of the residuals were checked to assess for linearity, homoscedasticity and normality; none of the plots indicated that these assumptions had been violated. For all regression models, multicollinearity among the independent variables was tested in two ways (Tabachnick & Fidell, 2007). First, correlations between the independent variables were inspected (see Table 3). Second, collinearity statistics (i.e. tolerance and variance inflation factors) and diagnostics (i.e. condition index and variance proportions) were conducted. None of these analyses suggested that multicollinearity was a cause for concern.

**Table 4.** Adjusted *R*-square and beta values for the regression analyses predicting psychological distress and QoL at baseline (*N *= 138).

Variable	Anxiety	Depression	Mental QoL	Physical QoL
Age	−.05	−.02	−.04	−.15
Identity	−.05	.01	.06	−.13
Timeline – chronic	.28*	.29*	−.15	−.30*
Timeline – cyclical	.01	.02	.03	−.09
Consequences	.00	.11	−.14	−.04
Personal control	−.09	−.05	.01	.14
Treatment control	.18	.07	−.01	−.24
Coherence	−.01	−.18	.04	.00
Emotional representations	.10	−.00	−.06	−.13
Risk causes	.00	.05	.04	.02
Psychological causes	.49***	.32**	−.55***	.14
Immunity causes	.06	−.05	−.09	−.10
Chance causes	−.01*	−.02	−.02	.02
Cardiac causes	−.04	−.29*	.20	.24
Gastrointestinal causes	−.07	.00	.12	−.14
Adjusted *R²*	.37**	.18**	.36***	.07

* *p *< .05

** *p *< .01

*** *p *< .001.

The variables included in the regression models explained 38% of the variance in anxiety, Adj. *R*²* *=* *.38, *F*(15,92) = 5.32, *p *< .001, 18% of the variance in depression, Adj. *R*²* *=* *.18, *F*(15,92) = 2.58, *p *=* *.003 and 36% of the variance in mental QoL, Adj. *R*²* *=* *.36, *F*(15,92) = 5.06, *p *< .001, but only 8% variance in physical QoL, Adj. *R*²* *=* *.08, *F*(15,92) = 1.59, *p *=* *.09. A stronger belief in psychological causes was significantly associated with poorer outcomes in all the analyses (with the exception of physical QoL), and a more chronic timeline was significantly associated with increased anxiety and depression and poorer physical QoL. A weaker belief in cardiac causes was associated with increased depression; however, the beta value was larger and in the opposite direction to the corresponding correlation coefficient, and is therefore likely be due to a suppressor effect.^1^. As a result, the significant beta value is not interpreted further.

### Associations with continued chest pain at follow-up

ANOVAs were conducted to examine whether baseline measures of psychological distress and illness representations were associated with continued chest pain (i.e. none, infrequent and frequent chest pain) at one-month follow-up, with subsequent Tukey *post hoc* analyses to assess between group differences (see Table 5). The three chest pain frequency groups did not differ in terms of illness representations; however, those with frequent pain at one month experienced higher levels of anxiety and depression and poorer mental and physical QoL at baseline than those with no pain at one month, and poorer mental QoL at baseline than those with monthly pain at one month (see Table 5). There were no significant differences between the chest pain frequency groups for any of the demographic variables.

**Table 5.** Means (SDs) and *F* values for psychological distress and QoL by chest pain frequency at follow-up.

Variable	Continued chest pain			*F*
	No pain *n* = 77	Infrequent *n* = 18	Frequent *n* = 39	
Anxiety	6.10_b_	6.33	8.28_a_	4.05^*^^,^^†^
Depression	3.92_b_	3.78	6.05_a_	4.53^*^^,^^†^
Physical QoL	49.75_b_	46.98	43.69_a_	5.79^**^^,^^†^
Mental QoL	45.89_b_	49.37_b_	40.52_a_	4.52^*^

* *p *< .05

** *p *< .01

*** *p *< .001.

^†^ Brown–Forsythe statistic (to account for homogeneity of variance).

Note: Means with different subscripts are significantly different from each other.

**Figure 1.**
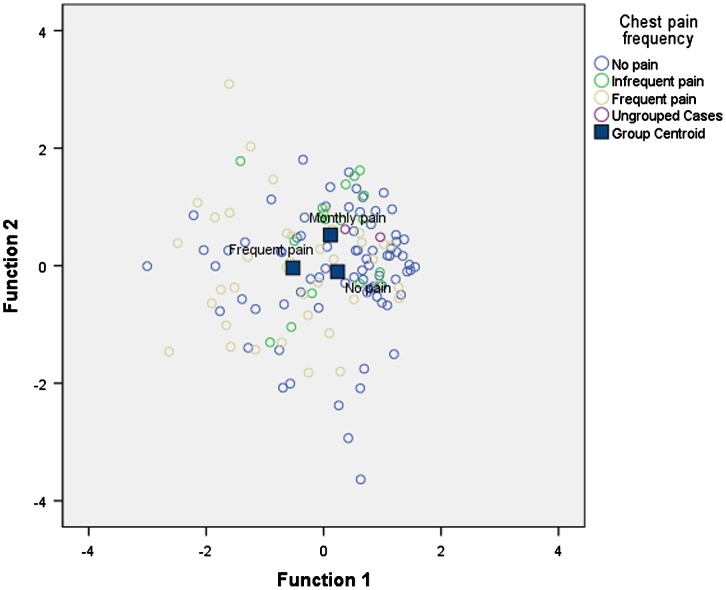
Combined plot of discriminant functions.

A discriminant function analysis was then performed to identify the key factors associated with continued chest pain. Discriminant function analysis is an extension of MANOVA that can be used to predict group membership from combinations of continuous independent variables. Baseline variables that were significantly related to chest pain frequency in the ANOVAs were included in the discriminant function analysis, i.e., anxiety, depression, and physical and mental QoL. Four cases were excluded due to missing data. The sample for the analysis therefore consisted of 134 participants (no pain *n *= 77, infrequent pain *n *= 18 and frequent pain *n *= 39). Two discriminant functions were calculated, which together significantly discriminated between the chest pain frequency groups, *χ²*(8) = 19.83, *p *<* *.001, although the second function alone was not significant, *χ²*(3) = 5.59, *p *=* *.13. As a result, only the first discriminant function is interpreted. The first function accounted for 11% of the total relationship between the predictors and group membership, Canonical *R*²* *= .11 and accounted for 72.5% of the between group variability. Examination of the combined plot of discriminant functions (see Figure 1) and the functions at group centroids indicated that function one primarily discriminated between those with frequent pain and the other chest pain frequency groups. The structure loading matrix was examined (see Table 6). Coefficients represent the unique contribution of each independent variable to the discriminant function; loadings >.50 were interpreted. The results of the discriminant function analysis indicated that anxiety, depression, and physical and mental QoL distinguished those with frequent pain from all other chest pain frequency groups, as they all loaded highly onto the first function. Thus, those experiencing frequent pain at one month were more likely to report more anxiety and depression and poorer physical and mental QoL at baseline than those with no pain or infrequent pain at one month.

**Table 6.** Summary of correlations of predictor variables with discriminant functions.

Predictor variable	Correlation of predictor variables with discriminant functions	
	1	2
Anxiety	−.70	−.05
Depression	−.73	−.20
Physical QoL	.83	−.32
Mental QoL	.67	.62

## Discussion

This study sought to examine the role of illness representations in explaining psychological distress and continued chest pain in patients with NCCP. Illness representations were associated with psychological distress and QoL at baseline, which, in turn, were related to continued chest pain one month later. Illness representations were not associated directly with continued chest pain. The results therefore provide only partial support for the CSM as illness representations were associated with psychological, but not physical, outcomes. Practically, the results suggest that both illness representations and psychological distress may be important targets for intervention in patients with NCCP as illness representations were associated with psychological distress and psychological distress was related to continued chest pain.

Levels of anxiety in the current sample were raised, in line with previous findings that psychological distress is common patients with NCCP (Webster et al., 2012; White et al., 2008). Depression was not significantly different from population norms, suggesting that anxiety may be more of a concern in this sample. Mental QoL was also impaired, further suggesting the psychological impact of NCCP. Physical QoL, however, was not impaired, suggesting that, for most participants, NCCP did not have a strong impact on patients’ physical activities, which is contrary to previous findings which have suggested that patients with NCCP feel the need to restrict their activity (e.g. Jerlock, Gaston-Johansson, & Danielson, 2005; Jonsbu, Dammen, Morken, & Martinsen, 2010). This discrepancy may be due to the acute nature of the sample as those with more chronic chest pain may find it more physically disabling.

Almost half of the patients were still experiencing chest pain at one month, with 28.7% experiencing frequent pain and 13.2% infrequent pain. This supports previous evidence indicating that patients with NCCP can experience persistent and continued pain following diagnosis (e.g. Eslick & Talley, 2008; Jonsbu et al., 2010; Ockene, Shay, Alpert, Weiner, & Dalen, 1980; Potts & Bass, 1993). Anxiety, depression and QoL, assessed at baseline, were found to be related to chest pain frequency one month later. This supports previous findings that psychological distress is associated with continued chest pain (Kisely, Guthrie, Creed, & Tew, 1997; White et al., 2008), and also highlights psychological distress as an important target for intervention. It has been suggested that NCCP may be caused by psychological disorders such as panic disorder (e.g. Beitman et al., 1989; Fleet et al., 1996); mediated through hypervigilance to cardiac-related sensations and heart-focused anxiety (Eifert, Hodson, Tracey, Seville, & Gunawardane, 1996; Eifert, Zvolensky, & Lejuez, 2000; White et al., 2010). Alternatively, psychological problems such as anxiety and stress may lead to chest pain more directly; for example, by causing increased muscle tension (Lundberg et al., 1994; Lundberg et al., 1999), oesophageal contractions (Anderson, Dalton, Bradley, & Richter, 1989) or chronic activation of the endocrine stress system (Fries, Hesse, Hellhammer, & Hellhammer, 2005). Introducing methods to reduce this anxiety may therefore be central to relieving NCCP.

There have been numerous previous psychological interventions for patients with NCCP, which have been effective in improving psychological outcomes (Kisely, Campbell, Yelland, & Paydar, 2012); however, these have often involved a number of sessions of Cognitive Behavioural Therapy. In settings such as EDs, it may be difficult to administer such interventions. Brief self-help interventions have previously been shown to be effective for anxiety disorders (Lewis, Pearce, & Bisson, 2012). Given the high level of anxiety in this sample, such interventions may also be useful for patients with NCCP. Furthermore, given anxiety in NCCP is related to increased health care use (Hadlandsmyth et al., 2013), interventions to improve anxiety may have implications for reducing health care costs. Brief information-giving leaflets for NCCP patients have also been shown to be effective in improving reassurance and reducing anxiety and depression (Arnold et al., 2009; Petrie et al., 2007). Incorporating anxiety-reduction techniques into such leaflets could further improve their effectiveness.

With regard to perception of causes, it is interesting to note that none of the mean ratings for the causes were above scale mid-points, suggesting that participants had weak perceptions of possible causes of their NCCP. However, more detailed analyses revealed gastrointestinal causes were strongly endorsed (i.e. a score of ≥4.00) by almost a third (31.2%) of participants. The next most frequently endorsed cause was chance (19.7%). All other causes were endorsed by less than 10% of the sample. These findings may, in part, be related to the timing of the baseline questionnaire, which was administered after patients received their NCCP diagnosis in an ED, which rules out a cardiac cause for the chest pain, but fails to provide a definitive alternative explanation. Gastrointestinal causes may be the next most obvious explanation, although they were unrelated to psychological distress, QoL or continued chest pain. Instead, participants who gave more weight to psychological causes had higher levels of psychological distress and poorer QoL, suggesting that some patients are aware of the potential link between anxiety and NCCP. Future research should seek to explore causal beliefs in patients with NCCP, using qualitative methodology, to examine this in detail.

The perception of psychological causes and a chronic timeline were related to elevated levels of psychological distress and poorer QoL, which, in turn, were related to continued chest pain. This supports previous findings that illness representations are related to psychological morbidity (Jonsbu et al., 2012; Schroeder et al., 2012). Illness representations may therefore be important targets for intervention to reduce initial levels of psychological distress, which may, in turn, reduce the experience of chest pain. The most important illness representation dimension for explaining increased psychological distress and impaired QoL was a belief in psychological causes. This relationship is in contrast to other research using the CSM which has found other dimensions such as timeline and consequences to be more important predictors than perceived causes (Hagger & Orbell, 2003). Furthermore, this relationship contradicts previous suggestions that patients may not acknowledge psychological explanations of NCCP and may therefore be resistant to engaging with psychological interventions (Esler & Bock, 2004; Risør, 2009). Considering the present findings, NCCP patients in ED might be more open to psychological therapies to manage their chest pain.

Patients who perceived their pain to have a more chronic timeline suffered more psychological distress and poorer QoL. This suggests that a pessimistic view about the chronicity of pain may lead to poor psychological outcomes. In other research, those who experience chronic (as opposed to acute) pain have been shown to report a more chronic timeline (Moss-Morris et al., 2002); moreover, timeline has previously been shown to have a strong relationship with psychological outcomes (Hagger & Orbell, 2003), including in MUS (Frostholm et al., 2007; Moss-Morris, 2005). Timeline may therefore be a target for intervention. However, if patients are given a way to cope with or reduce their pain, this may indirectly reduce perceptions of timeline.

The perception of psychological causes and a more chronic timeline were the only illness representation dimensions that consistently predicted psychological outcomes in this study. This is in contrast to previous research in NCCP, which has found that more of the dimensions to be predictive of outcomes, including identity, consequences, control and emotional representations (Donkin et al., 2006; Jonsbu et al., 2012; Schroeder et al., 2012). This difference may be due to the fact that previous studies have used the brief IPQ (Broadbent et al., 2006), which uses single items to assess each dimension. In addition, previous studies have not considered the impact of perceived causes of NCCP. Given NCCP patients do not receive a firm diagnosis of cause (merely a rule-out of cardiac causes), perception of cause may play a strong role in their adjustment. The present study highlights that perception of cause is a very important dimension in NCCP patients, and therefore could be a target for intervention. Furthermore, future research considering illness representations in NCCP patients should investigate perception of cause as a priority.

Although the present findings provide some support for the CSM, they also highlight an important limitation. The CSM proposes that illness representations are related to both psychological and physical adjustment (Leventhal et al., 1980); however, the present results suggest that the model may only be related to psychological, but not physical, outcomes. This is in line with a previous application of the CSM among patients with health problems and those with MUS, which have found that illness representations are more strongly related to psychological, rather than physical, outcomes (Frostholm et al., 2007). In addition, a meta-analysis of the CSM revealed stronger average correlations between illness representations and measures of psychological distress and well-being that with measures of physical outcomes and disease state (Hagger & Orbell, 2003). As such, the CSM may provide a better model of psychological, rather than physical, adjustment.

The conclusions of this study are tempered by a number of study limitations. First, there was significant attrition at baseline and follow-up, despite reminder phone calls to non-respondents. This may therefore have biased the findings towards those who might be more inclined to respond to questionnaires. High attrition at baseline was due to the majority of participants taking their questionnaire away, to be returned via freepost envelope. Receiving a non-cardiac diagnosis may have led some participants to no longer believe that their NCCP was as important issue. As a result, they may not have felt the need to respond to the questionnaire. Unfortunately, we were not able to compare responders vs. non-responders to the baseline questionnaire. However, non-responders at follow-up were younger and more likely to be male. In addition, they reported higher levels of anxiety and stronger perceptions of risk and psychological causes at baseline. These attrition biases limit the generalisability of the findings. The higher anxiety levels in non-responders may also suggest that issue of anxiety in NCCP may be greater than found in the present study, further supporting the need for intervention.

Second, participants were only recruited during the hours of 9 am and 7 pm, due to the fact that the specialist chest pain nurses only attended to patients during these hours (outside of these hours, patients were treated by ED doctors). This may therefore impact the findings, as it is possible that those who attend the ED outside of these hours may differ in terms of characteristics such as anxiety. However, patients who were discharged between 7 pm and 9 am were often asked to return to the ED during working hours for an exercise tolerance test, at which point they would have been screened for eligibility.

The final caveat is that the sample was recruited from one ED in the UK, which may further limit the generalisability of the findings. However, the age and gender profile of the sample reflected those reported in previous studies of acute care NCCP patients (see Webster et al., 2012).

## Conclusions

The current findings are consistent with previous research that has shown that patients with NCCP suffer poor psychological outcomes and impaired QoL and also confirm previous evidence that psychological distress is related to continued chest pain. The findings extend previous research by examining the role of illness representations in explaining both psychological distress and continued chest pain. Illness representations were related to psychological distress and QoL in line with research on other medical conditions but, contrary to the CSM, were not related to continued pain, despite some previous evidence suggesting that they are predictive of physical outcomes (Hagger & Orbell, 2003). Instead, psychological distress and QoL were related to continued chest pain. The most important illness representation dimension was belief in psychological causes in maintaining chest pain. This study therefore supports the use of low intensity interventions aimed at reducing anxiety in order to improve physical outcomes in patients with NCCP.

**Acronyms:**
ED: emergency department
NCCP: non-cardiac chest pain
QoL: quality of life
MUS: medically unexplained symptoms

## Conflicts of interest and source of funding

The research in this paper was conducted as part of the first author’s PhD, which was funded by an Economic and Social Research Council (UK) and Medical Research Council (UK) interdisciplinary studentship. There are no conflicts of interest.

## Note

1. In multiple regression analyses, a suppressor variable enhances the prediction of the dependent variable, not as a result of its own association with the dependent variable, but as a result of suppressing variance that is irrelevant to the prediction of the dependent variable. The presence of a suppressor effect is identified when (i) the absolute value of the correlation coefficient between an independent variable and the dependent variable is smaller than the absolute value of the corresponding beta coefficient and (ii) the correlation and beta coefficients have opposite signs (Tabachnick & Fidell, 2007) Both of these criteria were met in the present case given that *r *= .15 and *β *= −.29. Further analyses identified chronic timeline, coherence and psychological causes as potential suppressor variables as all three had correlation and beta coefficients that were consistent in size and direction. However, it was only when all three were removed from the regression equation that the beta coefficient for cardiac cause became non-significant (*β *= −.22, *p *= .07).
